# Emerging Optoelectronic Devices Based on Microscale LEDs and Their Use as Implantable Biomedical Applications

**DOI:** 10.3390/mi13071069

**Published:** 2022-07-04

**Authors:** Haijian Zhang, Yanxiu Peng, Nuohan Zhang, Jian Yang, Yongtian Wang, He Ding

**Affiliations:** 1 Beijing Engineering Research Center of Mixed Reality and Advanced Display, School of Optics and Photonics, Beijing Institute of Technology, Beijing 100081, China; haijianzh@163.com (H.Z.); yanxiup@foxmail.com (Y.P.); jyang@bit.edu.cn (J.Y.); wyt@bit.edu.cn (Y.W.); 2 GMA Optoelectronic Technology Limited, Xinyang 464000, China; ceo@gdgma.com

**Keywords:** microscale LEDs, lift-off, transfer printing, optogenetics, phototherapy, photometry

## Abstract

Thin-film microscale light-emitting diodes (LEDs) are efficient light sources and their integrated applications offer robust capabilities and potential strategies in biomedical science. By leveraging innovations in the design of optoelectronic semiconductor structures, advanced fabrication techniques, biocompatible encapsulation, remote control circuits, wireless power supply strategies, etc., these emerging applications provide implantable probes that differ from conventional tethering techniques such as optical fibers. This review introduces the recent advancements of thin-film microscale LEDs for biomedical applications, covering the device lift-off and transfer printing fabrication processes and the representative biomedical applications for light stimulation, therapy, and photometric biosensing. Wireless power delivery systems have been outlined and discussed to facilitate the operation of implantable probes. With such wireless, battery-free, and minimally invasive implantable light-source probes, these biomedical applications offer excellent opportunities and instruments for both biomedical sciences research and clinical diagnosis and therapy.

## 1. Introduction

Miniaturized, thin-film, flexible, and functional optoelectronic devices and systems have been developed thanks to advanced fabrication techniques in recent years [1,2,3]. These emerging designs are increasingly acceptable for biomedical applications due to their characteristics matching biological tissue features. Implanting these innovative optoelectronic devices and systems into biological tissues or organs can provide precision optical stimulation [4,5], high sensitivity detection [6,7,8], effective photon treatment [9,10], and other benefits. Furthermore, these devices and systems encourage novel biomedical concepts by utilizing remote control and wireless energy delivery technologies.

Organisms can be diagnosed or treated by stimulating the target site optically, electrically, and chemically, and by collecting relevant information on the potential, fluorescence, temperature, chemical composition, etc. Different from electrical stimulation and chemotherapy, the optical methods have the better spatial resolution, and target selectivity, is contact-free, etc., and has unique advantages in specific situations, such as closed-loop peripheral neuromodulation [11], visual recording of physiological signals [12], selective photochemical destruction of diseased cells [13], etc. The conventional optical lighting methods in biomedical research typically employ cumbersome light sources and combine them with fiber-optics for light delivery and collection in deep tissue [14,15]. The tethering concepts exacerbate tissue damage and restrict movement, resulting in challenges to evaluation and impeding trial reproducibility. Inorganic microscale light-emitting diodes (LEDs) are high-efficiency light sources that have the advantages of small footprint, high brightness, long life, and low power consumption, as well as easy integration with other electronic components [16]. By removing the grown bulk substrate and integrating thin-film microscale optoelectronic devices with flexible carrier substrates, these implantable optical probes can solve the aforementioned issues in contrast to conventional fiber-optics [6,17,18]. However, these implantable optoelectronic devices need a power system to realize their operation and functionality. Although the external battery could provide sufficient and stable power, the size, weight, and replacement are barriers to further minimization [19,20,21]. Wireless power harvesting strategies, e.g., ultrasound transduction, photovoltaic conversion, magnetic resonant coupling, and remote circuit control with Bluetooth wireless technologies, have been developed [2,3,22]. Thus, a fully implantable, wireless, battery-free, miniaturized optoelectronic lighting system allows for long-term implantation and biomedical research.

This review focuses on the recent state-of-the-art manufacturing for microscale thin-film devices and their integration with heterogeneous flexible substrates. Subsequently, we emphatically show several types of implantable optoelectronics used for optogenetic stimulation, local photometry, and phototherapy in biological tissue. Finally, we summarize wireless power supply techniques, which make these tetherless systems more biocompatible.

## 2. Microscale, Thin-Film, Heterogeneous Integrated Optoelectronic Semiconductor Devices

Bio-optoelectronics are ground-breaking technologies that not only address the fundamental mismatch between the properties of biomedical systems (soft, curvilinear, and transient) [23] and those of modern semiconductor devices [24] (rigid, planar, and everlasting), but also demonstrate exceptional capabilities in energy conversion [25,26], function display [27,28], wireless sensing [4,29,30], stimulation [31,32,33], and other areas [34,35]. Thin-film semiconductor devices, such as microscale thin-film LEDs and photodiodes, are typically removed from growing substrates and transferred to other substrates employing epitaxial lift-off and heterogeneous integration processes [36,37]. These technologies can successfully reduce device thickness and weight, retrieve the grown substrate, significantly expand functionalities, and enable a variety of emerging applications to become possible, including bio-integrated electronics [38,39], transient electronics [40], deformable optoelectronic devices [41,42], wireless implantable light sources for optogenetic stimulation [4,18], therapy [9,10], and in-situ photometry [8].

According to the light emission wavelength requirement, the semiconductor materials for LEDs typically are GaN, InGaN, GaP, GaAs, InP, etc., with the bandgap from ~3.4 eV to ~1.3 eV, corresponding to the luminescence from ultraviolet to infrared. The light penetration depth in tissue is highly related to the absorption and scattering, in which the light emission in a wavelength ranges from 650 nm to 950 nm as the first “biological transparency window” presents a deeper depth of ~1–2 cm [43,44]. The LED devices are grown via the metal organic chemical vapor deposition (MOCVD) method and fabricated with lithographic etching with sizes ranging from several hundred to a few (~10 μm) micrometers [45,46]. Moreover, the etching process can be cataloged into wet etching and dry etching processes, in which smaller feature sizes, deep etch undercuts, and high-aspect ratio features can be realized with the dry etching process, but large feature sizes, lower cost, and a faster process can be realized with a wet etching process.

**Figure 1:**
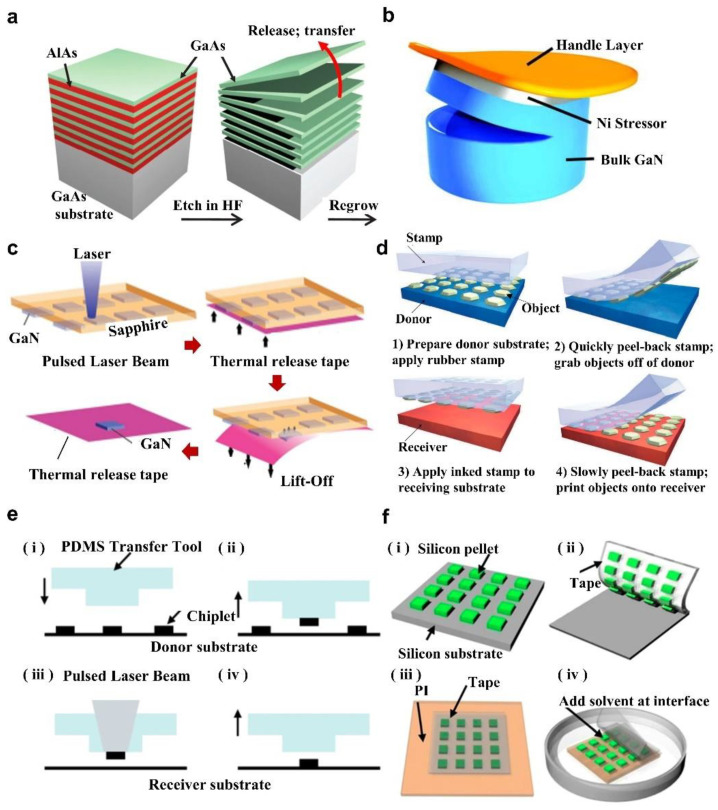
The heterogeneous integration with epitaxial lift-off techniques and representative transfer printing techniques. (**a**) Schematic illustration of the release of the thin GaAs layer by selective removal of the sacrificial layer based on the wet etching process. Reproduced with permission from Ref. [47]. Copyright 2010, Nature. (**b**) Schematic illustration of the controlled spalling process for bulk GaN substrates. Reproduced with permission from Ref. [55]. Copyright 2017, Journal of Applied Physics. The key step is to apply a flexible treatment layer to the surface of a tensile stress layer with a critical thickness to release the semiconductor film by creating cracks near the wafer edges and mechanically guiding the fracture front through the substrate. (**c**) Thin-film GaN-based LED is released from the grown sapphire substrate via the laser lift-off (LLO) process. Reproduced with permission from Ref. [52]. Copyright 2016, Applied Physics A. (**d**) Schematic illustration of the kinetically controlled transfer printing process using Polydimethylsiloxane (PDMS) stamps, where the peel speed of stamp pickup and printing is a significant determination for adhesion. Reproduced with permission from Ref. [60]. Copyright 2005, Nature Materials. (**e**) The laser-assisted PDMS transfer printing technique, which is selectively released by the device via laser-induced thermomechanical interface mismatch. (i) alignment; (ii) contact and retrieval; (iii) laser heating; (iv) separation. Reproduced with permission from Ref. [37]. Copyright 2018, Npj Flexible Electronics. (**f**) Schematic diagram of a solvent releasable tape for the transfer printing of an ultra-thin array of silicon pellets from a fabricated wafer onto a thin polyimide (PI) substrate by using a chemical solvent to adjust the interfacial adhesive strength of the tape. (i) Silicon pellet array fabrication; (ii) picking up; (iii) lamination; (iv) peeling off. Reproduced with permission from Ref. [65]. Copyright 2015, Scientific Reports.

Representative thin-film device fabrication techniques, including chemical lift-off (CLF) [47,48,49,50], laser lift-off (LLO) [51,52,53,54], controlled spalling technique (CST) [55,56], and other industrial-scale epitaxial lift-off procedures, have been developed to achieve the substrate-free devices. Chemical lift-off (CLF) is a process that involves selectively etching the sacrificial layer between the device layer and the growth substrate with chemical etchants. For example, hydrofluoric acid (HF) has been widely used to selectively etch AlAs sacrificial layers between the device structure layers and the GaAs substrate in the thin-film flexible GaAs-based IR epitaxial structure depicted in Figure 1a [47]. The material characteristics of the sacrificial layers must be suitable for the underlying substrate and overlaying device structures, and they can be selectively removed without damaging the device. Controlled spalling technology (CST) is based on a brittle fracture mode in which a tensile surface layer produces fractures parallel to (and below) the film/substrate contact. Figure 1b schematically illustrates the controlled exfoliation process [55]. The handle layer is critical because it promotes pure spalling mode fractures while reducing parasitic modes. A stressor layer of appropriate thickness can be constructed of various materials and created using techniques such as sputtering or electroplating. It can be carried out at room temperature using low-cost laboratory equipment in practically every stage of the semiconductor manufacturing process, from ingot casting to the formation of substrates to the completion of the product. Laser lift-off (LLO) separates epitaxial layer structures from transparent substrates, e.g., sapphire or SiC, using an excimer laser. Figure 1c depicts the laser selective stripping of GaN from a sapphire substrate, in which a high-energy pulsed laser beam penetrates the substrate and reaches the bottom surface of the GaN device [52]. The base interface absorbs photon energy and generates heat, and the GaN at the interface decomposes into liquid gallium at room temperature and N2, thereby leading to the thin-film GaN device separating from the sapphire substrate [51,52]. These epitaxial lift-off techniques mentioned above enable thin-film optoelectronic devices to be constructed as flexible, lightweight, and highly integrated systems [25,57,58,59].

The transfer printing methods pick up the released devices from the grown substrate and integrate them into the heterogeneous substrate, in which the representative carrier for the released devices are polydimethylsiloxane (PDMS) stamps [60,61,62], functional taps [63,64,65,66,67,68,69,70,71], and vacuum holders [72]. The adhesion between the released devices taken with the PDMS stamp carrier and the substrate is critical for the transfer printing process, as illustrated in Figure 1d, in which the stamp and device adhesion intensity to the substrate is proportional to movement speed [60]. The adhesion at the interface is tuned by high-speed coverage and low-speed peeling to enable the transfer of functional structures from donor substrates to acceptor substrates [61]. Transfer printing using PDMS stamps is very convenient and precise, but the transfer efficiency and multi-device success rate are poor, resulting in certain challenges for massive transfer processes [37,73]. In order to conquer these drawbacks, the laser-assisted PDMS has been recently proposed to update the capabilities of the transfer printing technique [73,74]. Figure 1e depicts the process flow of laser-assisted PDMS transfer printing, in which the separation of devices is accomplished by a significant thermomechanical mismatch in interface-driven delamination at a temperature over 275 °C due to the application of laser pulses [37,51]. Thanks to the high spatial resolution and patterned shape features of the laser, the high selectivity and multi-device transfer printing process can be realized. In addition to the PDMS stamp, the tapes with adjustable adhesives are also a good choice for transfer printing, such as soluble tape (ST) [63,64,65,66], thermal release tape (TRT) [67,68,69,75], and ultraviolet (UV) tape [70,71]. Figure 1f illustrates the tape transfer printing operation procedure schematically with acetone used as a binder conditioner [65]. The strong binding strength of the tape ensures highly reliable device pick up, whereas the introduction of solvents, temperature, or light during the printing process dramatically degrades the tape’s adhesion, guaranteeing the printing of devices on the receiving substrate.

The aforementioned epitaxial release and transfer printing process permits the creation of epitaxial layers of micro thin-film type devices, as well as the preparation of thin-film LEDs with good mechanical properties and steady operational performance coupled with flexible electronic devices, providing a solid foundation for the development of flexible, stretchable, and implantable biomedical tools for diagnosis and therapy.

## 3. Implantable LED Probes for Optogenetic Stimulations

Due to the strong scattering and absorption of light by biological tissues, particularly in the visible spectrum, it is challenging to effectively deliver light in deep biological tissues [76,77]. Although numerous exceptional materials, devices, and systems have been utilized, the direct injection of internal light sources to the desired location is more effective and reliable. In contrast to conventional fiber-optic implants, the implantable probe with microscale thin-film optoelectronic devices integrated on a flexible substrate, powered by a wireless energy design, and remotely controlled, exhibits excellent biocompatibility, no constraints, high precision stimulation, and sensitive detection. These implantable optoelectronics open up possibilities for robust biomedical routine applications, including applications in neurostimulation, phototherapy, and physiological monitoring.

**Figure 2:**
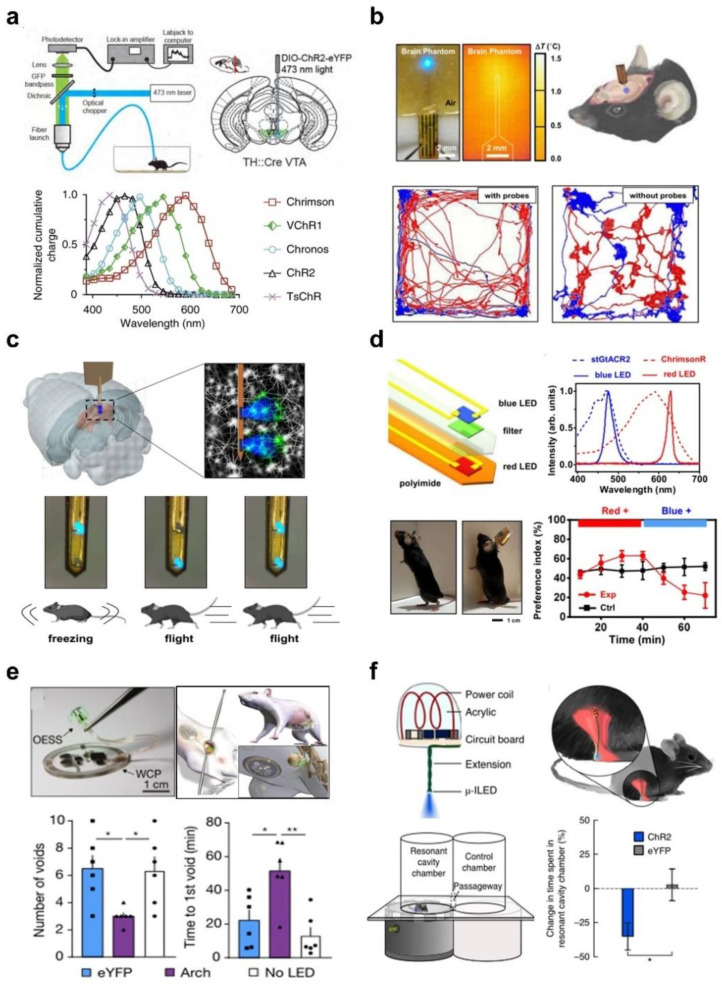
Implantable microscale LED probes for optogenetic neuromodulation via light delivery in biological tissue. (**a**) Schematic diagram of protein-expressing bioluminescence by optical fiber implantation in specific brain regions in a mouse model (top). Reproduced with permission from Ref. [79]. Copyright 2014, Cell. Different channelrhodopsins action spectra (bottom). Reproduced with permission from Ref. [80]. Copyright 2014, Nature Methods. (**b**) A 3D model of a freely moving mouse implanted with a wirelessly operable optogenetic probe, along with probe photographs and infrared imaging, and recorded activity traces demonstrating the results of optogenetic modulation of behavior in freely moving mice. Reproduced with permission from Ref. [17]. Copyright 2019, IEEE. (**c**) The spatially and temporally resolved LED probe design enables two-channel optogenetic stimulation to control motor behavior in mice exhibiting pronounced flight and aggression. Reproduced with permission from Ref. [18]. Copyright 2022, iScience. (**d**) A dual-color implantable light source probe for optogenetic stimulation realizes the bidirectional neural regulation of individual motor and social behavior preferences in free-moving mouse models. Reproduced with permission from Ref. [37]. Copyright 2022, Nature Communications. (**e**) A fully implantable wireless battery-free system for automated closed-loop optogenetic neuromodulation of peripheral nerves to modulate bladder function (n = 6, * *p* < 0.05, ** *p* < 0.01, two-way ANOVA). Reproduced with permission from Ref. [11]. Copyright 2019, Nature. (**f**) A miniaturized subdermal implant. Optogenetic stimulation with an implantable LED in the mouse sciatic nerve regulates motor behavior and achieves a significant preference for location (* *p* = 0.039, unpaired *t* test). Reproduced with permission from Ref. [81]. Copyright 2015, Nature Methods.

Different from the conventional electrical or chemical neuromodulation, optogenetics can precisely control and monitor neuronal cells by delivering light to channelrhodopsin-expressing neurons with fiber-optics [78,79]. As shown in Figure 2a, conventional optical fibers are implanted into the ventral tegmental area (VTA) of transgenic TH::Cre mice expressing channelrhodopsin-2, which delivered 473 nm excitation light to provide neuromodulation, and the fluorescence emission of calcium ion in this brain region is simultaneously collected to enable recording of neural activities [79]. Genetically engineered to express different light-sensitive opsin, channelrhodopsin expression is significantly dependent on different excitation wavelengths for optimal optogenetic stimulation. For instance, channelrhodopsin-2 (ChR2) is sensitive to blue light, while ChrimisonR is sensitive to red light [80].

Figure 2b depicts an implantable light source probe based on thin-film, microscale optoelectronic GaN-based LEDs on flexible copper/polyimide substrates for activating ChR2-expressing neurons in the brain with blue-emitting light [17]. The dimensions of the microprobe are ~300 μm in width, ~140 μm in thickness, and ~5 mm in length, and it is encapsulated in an epoxy composed of PMDS and paralyene. This implantable optical probe has Young’s modulus comparable to that of tissue, outstanding biocompatibility, negligible tissue invasion, and a safe temperature rise. This probe could be mounted on the mouse’s head without impeding its movement, thanks to the remote-control circuit design and lightweight battery supply. Compared to the control group, the movement paths of mice with implantable probes are uniformly distributed, and there is no effect on social behavior. To elicit more complex and diverse neuronal activity responses, multiple brain regions and different colors of excitation light are required, for which dual-channel and dual-color light-emitting implantable probes have been established in the subsequent research. As depicted in Figure 2c, the flexible implantable probe comprises two thin-film GaN micro-LEDs positioned at different depths (spacing, 0.6 mm) in the ChR2-expressing brain areas; these brain regions can be photostimulated separately. Under remote control, this miniature implantable probe delivered synchronous optogenetic stimulation to induce freeze and fly behaviors, respectively, by activating or inhibiting ChR2-expressing neurons at different locations in the superior colliculus with the LEDs in different locations [18].

Optogenetic stimulation of the same neurons expressing different light-sensitive proteins with correlated light enables the analysis of more complex and diverse neural activity responses, rather than optogenetic stimulation of neurons in different locations. As shown in Figure 2d, the co-expression of two channelrhodopsins in the same set of neurons facilitated efficient light-induced cellular depletion by controlling cation and anion fluxes and hyperpolarization under red and blue light stimulation, respectively [31]. The implantable light source probe with red and blue light emission comprises vertically integrated microscale thin-film InGaP and GaN LEDs with stacked dimensions of 125 × 180 × 120 μm^3^ and exhibits superior biocompatibility for long-term light stimulation in vivo. Based on the light emission intensity distribution of two LEDs, they can effectively excite the corresponding channel proteins without interfering with each other. The microscale LED probe can be implanted in the head of a freely moving mouse without hindering its movement, controlled by a lightweight head-mounted circuit. From the results of the mouse position preference experiment, it can be concluded that dual-color optogenetic stimulations on two spectrally distinct opsins (ChrimsonR and stGtACR2) in mouse models clearly demonstrate bidirectional excitation and inhibition capacities for individual and social preference and aversion.

In addition to the brain, optogenetic stimulation with implantable light source probes can be applied to modulate and treat other neural systems. Figure 2e depicts a wireless closed-loop system for optogenetic peripheral neuromodulation that combines physiological signal monitoring and photostimulation with real-time signal processing and modulation for closed-loop control of bladder dysfunction. The optoelectronic stimulation and sensing (OESS) devices consist of a stretchable, flexible strain gauge and a microscale LED emitting at a wavelength of 540 nm [11]. The module encircles the bladder to measure volume changes and subsequently provides optogenetic stimulation on the sensory afferent neurons that innervate the bladder. Urination times and frequencies measured in mice with bladder dysfunction are significantly lower in the experimental group (purple box) than in the control group (white and blue boxes). Thus, in the presence of bladder hypofunction, optogenetic stimulation of bladder contraction improves urination and restores bladder dysfunction in freely moving animals based on the comparison of the experimental and control groups. Figure 2f depicts an LED implanted in the spinal cord and peripheral nerve endings powered by high-frequency electromagnetic waves to perform optogenetic stimulations [81]. In the experimental setup depicted at the bottom of Figure 2f, the implantable LEDs are automatically activated in the resonant cavity, and the peripheral nerves of the ChR2-expressing mice are subsequently subjected to pain and aversion to the area due to optogenetic stimulation. Consequently, mice spend significantly less time in the resonant chamber than in the chamber without optogenetic stimulation. Therefore, implantable probes based on microscale LEDs are effective for optogenetic neuronal modulation.

## 4. Implantable LED Probes for Phototherapy

**Figure 3:**
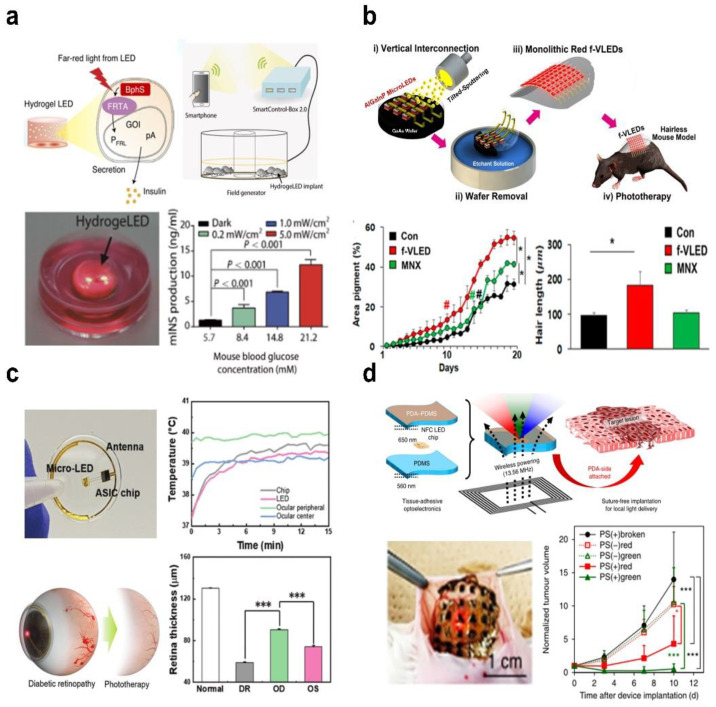
Microscale LEDs as implantable light sources for phototherapy. (**a**) A smart miniaturized wireless system for remotely controlling the release of glucose-lowering hormones from optically engineered cells in diabetic mice. BphS and FRTA, transcription regulators; GOI, genes of interest; pA, polyadenylation signals; P_FRL_, vector containing luciferase genes. Reproduced with permission from Ref. [82]. Copyright 2017, Science Translational Medicine. (**b**) Schematic illustration of trichogenic photostimulation by monolithic flexible AlGaInP vertical LEDs, with significantly improved hair-growth length and area after trichogenic treatments (**#** *p* < 0.05, two-way ANOVA; * *p* < 0.05, paired *t* test). CON, blank control group; MNX, minoxidil (a drug that stimulates hair growth). Reproduced with permission from Ref. [83]. Copyright 2018, ACS Nano. (**c**) Contact lens with a wirelessly powered LED for the treatment of diabetic retinopathy, with significant reduction in neovascularization and retinal hemorrhage following photobiomodulation therapy (*** *p* < 0.0005, paired *t* test). DR, diabetic retinopathy; OD, LED treatment; OS, without LED treatment. Reproduced with permission from Ref. [84]. Copyright 2022, Advanced Science. (**d**) Metronomic photodynamic therapy employs tissue-adhesive, wirelessly powered LEDs to activate photosensitizers and generate singlet oxygen for treatment (* *p* <  0.05, *** *p* <  0.001, Tukey’s honestly significant difference test). PS(+), add photosensitizer; PS(−), without photosensitizer. Reproduced with permission from Ref. [88]. Copyright 2019, Nature Biomedical Engineering.

As implantable light source probes are an efficient method of delivering light to deep tissues, light therapy with such a design is also attractive thanks to its stable and sufficient light energy and is critical for treating target lesions in vivo. Figure 3a depicts wirelessly powered LEDs implanted in diabetic mice, in which the 730 nm light activates photoreceptors (monophosphate synthase) and triggers insulin gene expression to rapidly restore steady-state blood glucose levels in freely moving mice [82]. To design custom receiver electronics compatible with external smart devices, the system customizes an implant structure called “HydrogeLED”, which incorporates engineered cells carrying alginate saline gel as well as wireless power coils and LEDs into a 15 mm spiral cylinder. This smart device further incorporates a blood glucose monitor, enabling semi-automatic blood glucose-dependent regulation via a wireless power transmitter that controls the LED lighting intensity and duration. This capability is demonstrated in diabetic mice implanted with the device, where the luminescent power of the LED is controlled in real-time to release different concentrations of insulin as the blood glucose concentration changes.

Figure 3b shows a wearable photon therapy concept for hair growth with a flexible, thin-film, high-performance 30 × 30 AlGaInP-based vertical μ-LEDs array, which is fabricated by the transfer-free monolithic interconnection of top n-electrode and bottom p-electrode [83]. The flexible μ-LED array can be used for wearable biomedical stimulation due to its high light output (up to 30 mW mm^−2^), low forward voltage (~2.8 V), and excellent flexibility. The red light with a wavelength of 650 nm penetrates deep into the skin and stimulates hair follicles located 2 mm below the surface of the dermis. Based on in vivo experimental findings, it indicates that red light from f-VLEDs can successfully accelerate the Wnt/β-catenin signal, hence promoting hair follicle cell proliferation and enhancing mice dorsal hair growth compared with the control group (black lines and boxes) and the medication group (green lines and boxes). Furthermore, the mechanical reliability is confirmed by periodic bending and unbending up to 10,000 times, and the maximum skin temperature is maintained below 40 °C without causing thermal damage to the skin when the radiative density of the LED is set to 1−5 mW mm^−2^. Figure 3c illustrates a non-invasive smart wireless luminescent contact lens with a microscale LED emitting far-red/near-infrared light (630–1000 nm) to perform recurrent photon treatment for diabetic retinopathy [84]. The smart contact lens combines LEDs with integrated circuit chips, wireless power, and communication systems on a PET film thermally cross-linked with a silicone elastomer. The temperature of each module is lower than 40 °C in the working condition based on the temperature versus time results, indicating no additional thermal damage. In the rabbit eye treatment experiment, the retinal thickness under the irradiation of the smart LED contact lens is 90.63 μm compared to 74.11 μm for the untreated one, demonstrating that the therapy effect on retinal damage is significantly improved. Due to the integration of optoelectronic devices into the contact lens and direct contact with the eye, the chip and LED raise the temperature to approximately 39 °C, but the temperature of the rabbit’s eye is between 38 °C and 40 °C, so no corneal damage is observed.

Photodynamic therapy (PDT) is effective phototherapy that employs the singlet oxygen produced by the interaction of light, photosensitizers, and oxygen, displaying high selectivity and minimal toxicity [85,86,87]. Figure 3d depicts a wireless optoelectronic system for photodynamic cancer therapy, in which red, green, and blue LEDs with peak wavelengths of 630, 530, and 460 nm are implanted under the skin to locally activate the photosensitizer to kill the tumor [88]. To reduce excitation light power losses, the device is encapsulated with polydopamine (PDA)-modified and unmodified PDMS nanosheets (600 nm thick) for perfect tissue adhesion. The photodynamic therapy with the implantable LED devices has significant antitumor effects compared to all control groups (PS(−) red, PS(−) green, and PS(+) broken) in vivo experiments.

## 5. Photometric Measurements Using Implantable LEDs and Photodetectors

**Figure 4:**
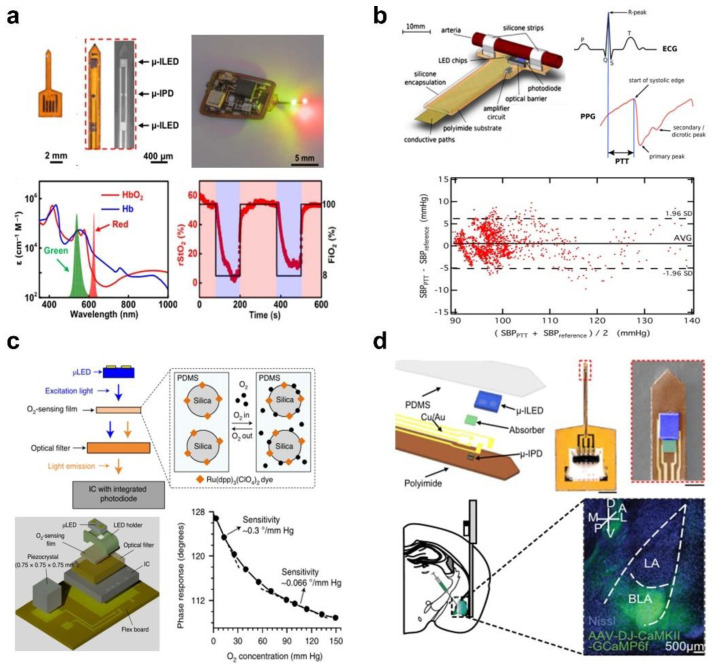
Implantable optoelectronic devices for physiological recording based on the optical response. (**a**) Miniaturized optoelectronic oximetry system with microscale LED and photodiode to detect the local hemoglobin dynamics in the deep mouse brain. The absorption spectra for HbO_2_ and Hb, with green and red shaded areas representing the emission spectra of LEDs. Estimated rStO_2_ (red traces) in the tissue region of anesthetized mice exposed to oxygen changes (black traces) between 100% (red blocks, ~100% O_2_ and 2% isoflurane) and 8% (purple blocks, 8% O_2_). Reproduced with permission from Ref. [89]. Copyright 2019, Science Advances. (**b**) Implantable system for long-term monitoring of blood pressure based on pulse transit time, and these data demonstrate a strong connection between systolic blood pressure measured by a commercial instrument and PTT measured by an optical sensor system. SBP, systolic blood pressure; SD, standard deviations; AVG, average. Reproduced with permission from Ref. [90]. Copyright 2013, Biomedical Microdevices. (**c**) Schematic illustration of a deep tissue O_2_ sensor by means of a biocompatible oxygen-sensitive film excited by an LED and its photoluminescence emission dependent on O_2_ concentration collected by an optical detector. Reproduced with permission from Ref. [91]. Copyright 2021, Nature Biotechnology. (**d**) Wireless optoelectronic photometry with a 473 nm blue LED and a filtered covered photodiode for monitoring Ca^2+^ dynamics in the basolateral amygdala (BLA) region of the brain expressing AAV-DJ-CaMKII-GCaMP6f (highlight with green color). Reproduced with permission from Ref. [6]. Copyright 2018, PNAS.

Combining microscale LEDs and photodetectors as implantable photometry designs for real-time monitoring of human physiological signals yields new instruments for biomedical research and clinical diagnosis and treatment, including monitoring of blood oxygen saturation [7,89], blood pressure [90], calcium ion (Ca^2+^) dynamics [6,8], and tissue oxygenation [91]. Figure 4a illustrates an implantable oximetry probe for continuous recording of local hemoglobin dynamics in the deep brain, which consists of green and red LEDs with emission wavelengths of 540 nm and 625 nm, and a photodetector with a high spectral response (external quantum efficiency (EQE), ~74% at 540 nm and ~82% at 625 nm) [89]. With this difference in optical properties, the regional tissue oxygen saturation (rStO_2_) can be calculated by comparing the attenuation of red and green light in tissues with varying amounts of oxyhemoglobin (HbO_2_) and hemoglobin (Hb). After implantation in the deep brain, blood oxygen levels in the brains of freely moving mice can be continuously monitored with precise control over the proportion of inhaled O_2_ in a hypoxic chamber.

Figure 4b depicts the blood pressure calculated from the correlation of pulse transit time (PTT) at peripheral cardiovascular sites, which is generally defined as the time difference between the R peak of the electrocardiogram (ECG) and the onset of the systolic blood pressure slope of the photoplethysmography (PPG) [90]. The soft and implantable blood pressure monitoring system that combines a micro-LED and a photodetector can prevent the risk of blood pressure sensor implantation in blood vessels leading to thrombosis and the risk of vascular deformation of arteries resulting from the pressure of the extravascular system. As depicted in Figure 4b, the sensor incorporates 8 LEDs with a wavelength range from 590 nm to 1300 nm and a photodetector. By placing two sensors on both sides of the blood vessel, it is able to simultaneously measure the reflected and transmitted signals at the same time and calculate high-precision photoplethysmography and electrocardiogram signals. With this unique sensor system, a strong correlation between systolic blood pressure and pulse transit time can be discovered, supporting the high accuracy of the LED-based measuring method in clinical applications and establishing the groundwork for future clinical applications.

Tissue oxygen variations are highly correlated with physiological activities, such as neural activity, tissue perfusion, tumor microenvironment, and wound healing, and represent critical indicators of the operating status and functional integrity of tissues and organs [92,93]. Blood oxygen content is typically used to indirectly calibrate tissue oxygen saturation via the correlation between blood and tissue oxygen saturation. As depicted in Figure 4a, it is hard to accurately reflect the oxygen content in local tissues, especially in particular situations. Figure 4c shows a miniature implant for monitoring O_2_ in deep tissue powered by piezoelectric energy from ultrasound transduction, in which the implant contains an oxygen-sensing membrane excited by the microscale LED and a luminescent sensor for detecting the emission [91]. The biocompatible oxygen-sensitive film is composed of polydimethylsiloxane (PDMS) and ruthenium fluorescent dye silica particles. The interaction of oxygen alters the luminescent properties of the fluorescent material, providing feedback on changes in oxygen concentration in the range of 3.8–150 mm Hg, and is more sensitive in the low oxygen concentration range of 3.8–30 mm Hg compared to the rest range.

The neural activity of freely moving animal models provides vital insights into brain functioning and guides investigations of animal behavior. Figure 4d schematically shows the recording of neuronal activity employing genes expressing calcium indicators (GECI), which is a standard fluorescent technique that detects Ca^2+^ changes associated with cell activity [6]. The photometry consists of a microscale LED (270 × 220 × 50 μm^3^) and an adjacent photodetector (100 × 100 × 5 μm^3^) with a photolithographically defined narrow-band absorber (140 × 140 × 7 μm^3^) as an optical filter. Implantable photometry can detect transient calcium ion changes from active neurons in the basolateral amygdala (BLA) of the mouse brain, demonstrating the capability to monitor neural activity in freely moving animals. This implantable excitation light source based on microscale LEDs, in collaboration with a photodetector for measuring transmission, reflection, and emission, facilitates the development of minimally invasive photometers for applications including pulse oximetry, pH, and CO_2_ sensors.

## 6. Wireless Energy Supply Strategies for the Implantable Devices

**Figure 5:**
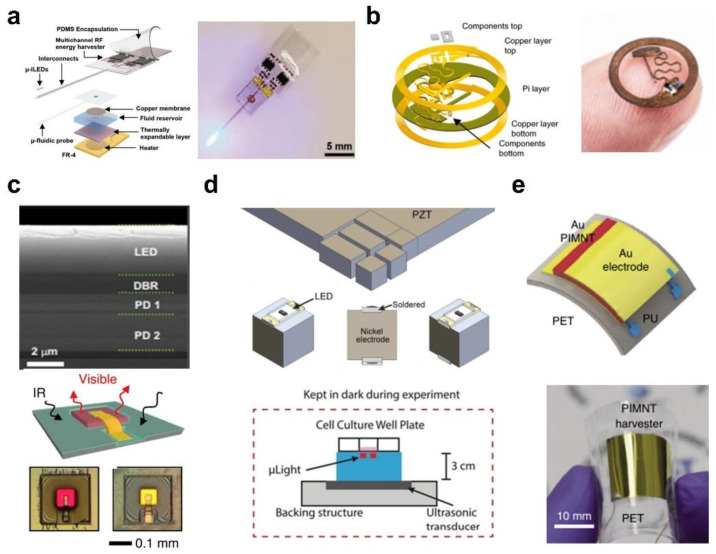
Wireless power transfer strategies for implantable and battery-free devices. The miniaturized implantable and wearable optoelectronic devices are powered by the following: (**a**) the subcutaneous coils based on far-field radiofrequency. Reproduced with permission from Ref. [95]. Copyright 2018, Small. (**b**) Magnetic resonant coupling power delivery. Reproduced with permission from Ref. [4]. Copyright 2018, Nature Electronics. (**c**) Photovoltaic conversion of near-infrared light. DBR, distributed Bragg reflector; PD, photovoltaic diodes. Reproduced with permission from Ref. [25]. Copyright 2018, PNAS. (**d**) An ultrasonic transducer. Reproduced with permission from Ref. [9]. Copyright 2019, Scientific Reports. (**e**) A flexible piezoelectric energy harvester. PET, polyethylene terephthalate; PIMNT, Pb(In_1/2_Nb_1/2_)O_3_–Pb(Mg_1/3_Nb_2/3_)O_3_–PbTiO_3_; PU, polyurethane. Reproduced with permission from Ref. [99]. Copyright 2021, Nature Biomedical Engineering.

Convenient, wireless, continuous energy supply is an enormous obstacle to the miniaturized and implantable devices for physiological signal recording and neurostimulation systems. The conventional power supply solutions with carrying or implanted batteries eliminate the range limitation of motion compared to cables, but the weight of the battery and the surgical infection risk of implantable batteries are the facing challenges [6,17,18]. Under these circumstances, power supply solutions with miniaturized, wireless, battery-free, and subcutaneous implantation operations in free-behavior animal models have been developed [2,3,94], and several recently reported wireless energy transfer methods are summarized in Figure 5.

As depicted in Figure 5a, the device integrates a microscale inorganic LED and drug delivery system, as well as a tiny, scalable multi-channel radiofrequency antenna for wireless energy transfer [95]. The antenna strategies, based on electromagnetic waves with frequencies ranging from 420 MHz to 2.4 GHz, are employed for long-distance power transmission via far-field electromagnetic waves, but the efficiency is highly dependent on the relative orientation of the antenna pair, interference and standing waves caused by reflections from environmental obstacles, and electromagnetic absorption leading to heating in tissue [96]. Near-field magnetic resonance coupling technology eliminates these limitations. As shown in Figure 5b, the device combines electronics with a flexible substrate material to capture energy by designing a high-quality inductor coil and voltage control via a power management module to drive a microscale LED probe for wirelessly implantable optogenetic stimulation [4]. The wireless power transmission technology employs transmit and receive resonators, which can significantly improve power transfer efficiency and distance, as the resonators are tuned to produce strong magnetic resonance coupling, thereby removing the phase limitation between the energy transmitter and receiver [94,97]. Meanwhile, this wireless power supply technology has a low operating frequency (from about 100 kHz to 200 MHz), which drastically reduces the absorption in biological tissues, thus providing greater penetration depth and minimizing adverse biological effects.

As depicted in Figure 5c, a thin-film microscale device achieves upconversion from near-infrared (~810 nm) to visible light (630 nm (red) or 590 nm (yellow)). The encapsulated microscale device can be implanted in the subcutaneous tissue and provides stable, long-term implantable light sources in behavioral animals [25]. Because near-infrared light penetrates biological tissues to a certain depth, photovoltaic cells also can absorb photons and provide solar energy to power the system. With the aid of advanced fabrication techniques, it is possible to fabricate ultra-small implantable devices with high spatial resolution and achieve high integration with implantable devices [22,76]. The ultrasonic energy transfer method shown in Figure 5d can also achieve a long-distance energy supply, in which the ultra-small device integrates a microscale LED into a piezoelectric crystal and can receive ultrasonic waves to generate energy to drive the LED for photodynamic therapy [9]. However, slight misalignment between the transducer and the implanted receiver reduces the coupling efficiency significantly. Additionally, the ultrasound sound generator must be in contact with the skin in order to reduce impedance mismatch at the tissue-air interface and keep the ultrasound path away from the bone region [98]. As shown in Figure 5e, a flexible piezoelectric device is able to flex and generate pulses of electricity to stimulate the motor cortex of mice, acting as a self-powered device for deep brain stimulation [99]. The piezoelectric effect that converts mechanical energy to electrical energy is a highly efficient and attractive method for self-powering without the requirement of external excitation. In order to generate sufficient power to satisfy the operation of the circuits, it is necessary to design complex circuits and develop high-performance piezoelectric materials, which are also incompatible with static conditions [100,101]. 

Wireless power strategies have a substantial impact on device functionality and performance. According to application scenarios and operating systems with different power consumption, such as optogenetic stimulation [94], electrical stimulation [102], and electrical recording [103], different power supply systems must be adapted. For instance, photometric recordings require several milliwatts of power [6,89], but electrophysiological recordings require only a few microwatts per channel [103,104]. Incorporating wireless data transfer technologies (Bluetooth [105], NFC [106], etc.) will eventually realize the remote signal transfer and wireless system operation.

## 7. Conclusions and Perspective

In conclusion, we outline the recent progress of the wireless optoelectronic design based on thin-film microscale LEDs, which exhibit great promise for neuroscience research and biomedical diagnosis and therapy in freely moving animals. Thin-film microscale LEDs are essential components of minimally invasive wireless optical response design, in which the released thin-film microscale LEDs are transferred and integrated onto biocompatible flexible substrate materials through specialized lift-off and transfer printing techniques. Taken together with the advanced wireless electronics and energy delivery mechanisms, they thereby enable battery-free, wireless implantable medical devices. This highly integrated, miniaturized wireless system can operate during extended periods of time in freely moving animals, allowing for the real-time, low-disturbance monitoring and regulation of physiological processes. These unique implantable optoelectronic devices exhibit the potential to be of great significance not only for optically based physiological research, but also for the clinical use of low-invasive, portable optical-biological interface devices.

Despite these remarkable achievements, there are critical challenges and abundant opportunities for these thin-film microscale LEDs for biomedical applications. The first is the chronic in vivo application of ultra-thin, ultra-soft, super stability LEDs with biocompatible characteristics that match the properties of the tissue. The second is large-range and automated communications, such as Internet of Things technology, which will facilitate multi-channel automated studies of large groups of animals simultaneously. The third is achieving reliable and efficient wireless energy transfer strategies, which allow devices and systems to operate efficiently and conveniently in complex environments. Last, the microscale LEDs are combined with various sensors (such as electrophysiological sensors, pressure sensors, temperature sensors, etc.) to expand the functionality, realizing closed-loop systems for detection, sensing, stimulation, and treatment. Consequently, these miniaturized implantable optoelectronic devices will provide unique capabilities as cutting-edge instruments and have a profound effect on scientific research and clinical therapy.

## Data Availability

Not applicable.
